# Can an EASYcare based dementia training programme improve diagnostic assessment and management of dementia by general practitioners and primary care nurses? The design of a randomised controlled trial

**DOI:** 10.1186/1472-6963-8-71

**Published:** 2008-04-02

**Authors:** M Perry, I Drašković, T van Achterberg, GF Borm, MIJ van Eijken, PL Lucassen, MJFJ Vernooij-Dassen, MGM Olde Rikkert

**Affiliations:** 1 Department of Geriatric Medicine of Radboud University; Nijmegen Medical Centre, Nijmegen, The Netherlands; 2 Centre for Quality of Care Research; of Radboud University; Nijmegen Medical Centre, Nijmegen, The Netherlands; 3 Department of Epidemiology and Biostatistics; Radboud University; Nijmegen Medical Centre, Nijmegen, The Netherlands; 4 Department of General Practice, of Radboud University; Nijmegen Medical Centre, Nijmegen, The Netherlands

## Abstract

**Background:**

Early diagnosis of dementia benefits both patient and caregiver. Nevertheless, dementia in primary care is currently under-diagnosed. Some educational interventions developed to improve dementia diagnosis and management were successful in increasing the number of dementia diagnoses and in changing attitudes and knowledge of health care staff. However, none of these interventions focussed on collaboration between GPs and nurses in dementia care. We developed an EASYcare-based Dementia Training Program (DTP) aimed at stimulating collaboration in dementia primary care. We expect this program to increase the number of cognitive assessments and dementia diagnoses and to improve attitudes and knowledge of GPs and nurses.

**Methods:**

The DTP is a complex educational intervention that consists of two workshops, a coaching program, access to an internet forum, and a Computerized Clinical Decision Support System on dementia diagnostics. One hundred duos of GPs and nurses will be recruited, from which 2/3 will be allocated to the intervention group and 1/3 to the control group. The effects of implementation of the DTP will be studied in a cluster-randomised controlled trial. Primary outcomes will be the number of cognitive assessments and dementia diagnoses in a period of 9 months following workshop participation. Secondary outcomes are measured on GP and nurse level: adherence to national guidelines for dementia, attitude, confidence and knowledge regarding dementia diagnosis and management; on patient level: number of emergency calls, visits and consultations and patient satisfaction; and on caregiver level: informal caregiver burden and satisfaction. Data will be collected from GPs' electronic medical records, self-registration forms and questionnaires. Statistical analysis will be performed using the MANOVA-method. Also, exploratory analyses will be performed, in order to gain insight into barriers and facilitators for implementation and the possible causal relations between the rate of success of the intervention components and the outcomes.

**Discussion:**

We developed multifaceted dementia training programme. Novelties in this programme are the training in fixed collaborative duos and the inclusion of an individual coaching program. The intervention is designed according to international guidelines and educational standards. Exploratory analysis will reveal its successful elements. Selection bias and contamination may be threats to the reliability of future results of this trial. Nevertheless, the results of this trial may provide useful information for policy makers and developers of continuing medical education.

**Trial registration:**

ClinicalTrials.gov ID NCT00459784

## Background

### Diagnosing dementia in primary care

Dementia is an increasing challenge for health care and social systems in developed countries. In Europe, 6.4% of the elderly over 65 years suffer from dementia, with an increase from 0.8% in the group aged 65–69 years to 28.5% at age 90 years and older. The total number of dementia patients in Europe is expected to increase from 7 million in 2000 to over 16 million in 2050 [1]. Currently, dementia seems to be under-diagnosed [2-4]. More than 50% of dementia patients living in the community have not been diagnosed by a GP or specialist [5,6]. In the Netherlands, detection of dementia takes place around one year before admission to a nursing home and three years before death [7], which is rather late.

Early diagnostic evaluation of patients possibly suffering from dementia is beneficial for both patient and caregiver. Reversible causes of dementia will be identified and treated timely. Formal disclosure of dementia diagnosis allows patients and carers to make future plans and provides early access to support services [8]. These actions may prevent or decrease psychological distress in patients [9]. Early education and support for caregivers facilitates adjustment over a longer period of time; it prevents crisis situations and delays nursing home admissions [10,11]. Non-pharmacological interventions, such as psychosocial interventions (10;11) and occupational therapy [12], have been shown to play a key role in dementia management. Pharmacological treatment may primarily be beneficial when started in the early stages of dementia. However, drugs that are currently available have only moderate effect on cognitive symptoms and ADL [13]. In the near future, disease-modifying drugs might become available since they are already subject to phase two and three trials.

Despite the acknowledged benefits of early diagnosis of dementia, both patients and doctors are reluctant to initiate cognitive assessment. Patient-related delay in early recognition of dementia is often caused by lack of insight into their condition, their view of memory loss symptoms as being normal for their age and fear of the negative consequences of dementia diagnosis [4]. GPs report that their own lack of knowledge and skills in diagnosing and treating dementia prevents them from starting diagnostic work-up in the early stages of dementia [14]. Other GP-related barriers include the absence of clear diagnostic guidelines and reliable, user-friendly screening tools, lack of time, of financial reward, of adequate resources such as access to neuropsychological consultations and neuro-imaging investigations, and lack of prescription right for cholinesterase inhibitors [15]. Thus, many GPs are sceptical about the benefits of early diagnosis, because they feel they have little to offer dementia patients and their caregivers [14,16,17]. Moreover, disclosure of diagnosis is considered to be difficult, because it may negatively influence patient-doctor relationships and take away patients' and caregivers' hopes [18,19]. GPs also fear the risk of diagnostic errors. Evidence of under-recognition of dementia has also been shown in primary care nurses for similar reasons [20]. Also, collaboration between primary care nurses and GPs is not very well developed, although dementia diagnosis and management may profit from it. This lack of collaboration is caused by conflicting expectations, domain discussions and poor coordination of care [21].

### Developing a Dementia Training Program (DTP) for primary care providers

To improve early detection in primary care, guidelines on dementia diagnosis and management were developed in several countries [22-25]. A multifaceted implementation program in Denmark did not show effects on adherence to practice guidelines [26]. Research teams in the UK and USA also developed educational programmes for implementation of their national guidelines. These programmes were successful in raising the rate of early dementia diagnosis and improving professionals' knowledge [27,28]. However, they showed only minor improvement in attitudes of health care providers regarding dementia diagnosis and regarding management of dementia in primary care [29,30]. Implementation strategies in these programs included the use of small group sessions, a Computerized Clinical Decision Support system (CDSS) [27], and internet support [28,29].

The educational programs in the studies discussed above primarily focused on GPs. However, dementia care management performed by collaborative interdisciplinary teams was found to be more effective in improving the quality of care than that performed either by GPs or primary care nurses [31]. Therefore, we designed a Dementia Training Program (DTP) for collaborative duos of GPs and primary care nurses, focused on teaching them how to share tasks in early diagnosis and management of dementia. Another unique component of our DTP is individual coaching, which has shown promising results in modifying behaviour of health professionals [32]. Furthermore, our Dementia Training Program is a multifaceted program [33] and consists, in addition to individual coaching, of two small-group interactive workshops [34,35] and a Computerized Clinical Decision Support System.

### Objectives

The objective of this paper is to describe the design of a randomised controlled intervention study, aimed at determining the effectiveness of a multifaceted Dementia Training Program (DTP) for general practitioners and primary care nurses, based on current national guidelines. We expect the DTP to improve professional performance in dementia diagnostics and disease management and GPs' and nurses' attitudes and knowledge regarding dementia.

## Methods

### Study design and setting

**Figure 1:**
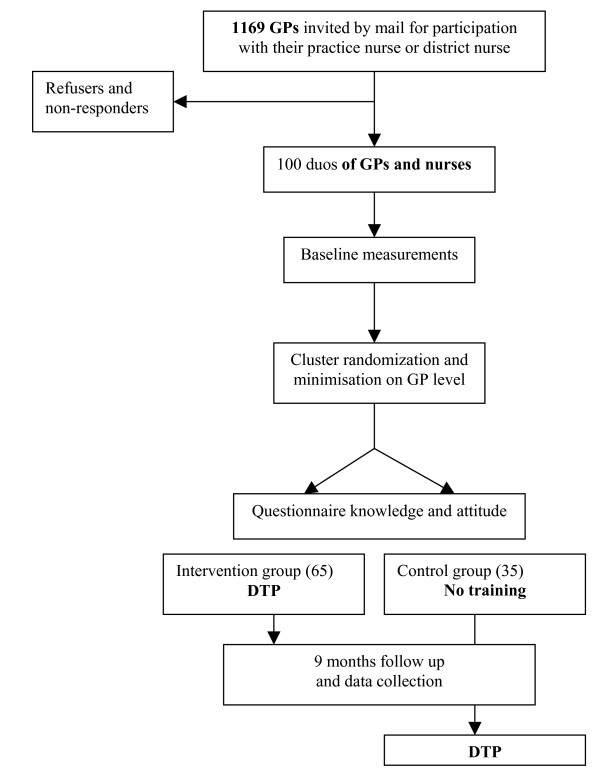
Procedure of recruitment and randomization.

This study is an outcome assessor-blinded randomised controlled trial. A cluster-randomised design will be used to compare duos of GPs and nurses (Figure 1).

### Study population

We plan to recruit 100 duos of GPs and practice or district nurses in the province of Gelderland, the Netherlands. We will approach all general practitioners in this province by mail and ask them to participate in the study. Participating GPs may choose to cooperate with their own practice nurse or with a district nurse. All GPs and affiliated nurses within the county are eligible for this study.

Frail elderly people, suspected of suffering from cognitive problems are the target group to be diagnosed and treated according to Dutch dementia guidelines. [22,25]. From this target group additional data on satisfaction and informal carer burden will be obtained. Informed consent will be obtained from patients and from their legal representatives. The Local Medical Ethical Committee, Commissie Mensgebonden Onderzoek Regio Arnhem – Nijmegen, concluded that this study did not include experiments with patients and therefore did not need to be tested for approval.

### Bias control and randomisation

Randomisation will take place after measurement of baseline data. Duos of GPs and nurses will be randomly allocated to one of the two conditions: 1. Dementia Training Program (DTP) and 2. no training at all; control group. (Figure 1). Randomization will be concealed; a person who is not responsible for recruiting subjects and has no knowledge of the study conduct will perform it on a computer.

Cluster-randomisation will be performed in order to avoid contamination by the effects of possible exchange of information within a cluster. A cluster was defined as all GPs working in the same practice or as all GPs working together with the same nurse.

In order to assure an equal distribution of baseline characteristics, duos will be randomised with adaptive weights regarding cluster size (one vs. more than one GP), age, sex, high or low percentage of elderly patients in practice (< or > 15%), practice location (rural or urban area) and nurse affiliation (district or practice).

Adherence to guidelines will be assessed by two independent researchers. In order to exclude the possibility of detection bias, these researchers will be blinded to the outcome of randomization.

### Intervention: Dementia Training Program (DTP)

**Figure 2:**
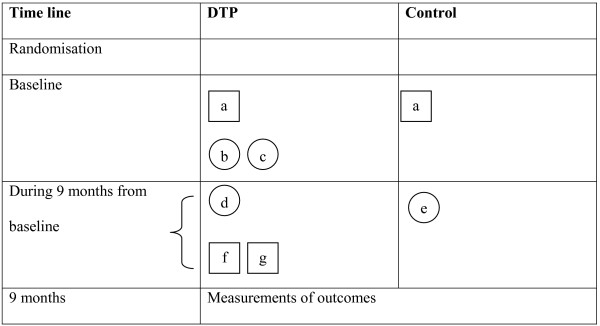
**Graphical depiction of the interventions of this trial on improving primary dementia care**. **a)** Dutch Dementia Guidelines for general practitioners and for community nurses. **b)** Workshop on dementia diagnosis and management for GPs and nurses as a duo. **c) **Coaching of GPs and nurses on dementia diagnostics and management in daily practice according to national guidelines and using the EASYcare assessment. **d)e)** Usual dementia care performed by GPs without coaching. **f)** Availability internet forum for patient questionnaires (MMSE, GDS, EASYcare assessment), discussion with colleagues, additional literature. **g)** Availability Computerised Clinical Decision Support System on dementia diagnosis.

DTP consists of two workshops and an individual coaching programme, including case-based consultation either by phone or by e-mail. Participants have access to an internet forum for discussion with colleagues, additional literature and individual training on dementia diagnosis and management. A Computerised Clinical Decision Support System on dementia diagnostics and management will be available to support GPs in decision making in daily practice. The content of the DTP and the CDSS is based on the recently published, evidence-based Dementia Guideline for Primary Care [36]. The DTP is graphically presented in Figure 2[37].

Methods used in the DTP to stimulate collaboration between GPs and nurses are the following:

1. Training of collaborative performance of geriatric function assessment according to dementia guidelines, using the EASYcare assessment. This assessment of geriatric function is carried out by a nurse and interpreted by a GP. Therefore, it requires collaboration in order to be performed.

2. Collaboration and training in fixed duos.

3. Training in collaborative workout of a dementia case assignment.

4. Presentation and discussion of 'task sharing' between GPs and nurses in dementia diagnostics and management.

5. Availability of a Dementia Guideline for Primary Care, which has exclusively been written for this project, based on evidence-based Dutch guidelines [22,25] and recent studies investigating the diagnosis and management of dementia in primary care. This Dementia Guideline for Primary Care contains a flow chart and recommendations regarding 'task sharing' and consultation moments between GPs and nurses.

#### Workshops

Two workshops were developed according to approved educational standards [38]. The first workshop was designed for both GPs and nurses to train collaboration in diagnosis and management of dementia. During the second workshop, nurses and GPs will be educated separately. The second workshop for GPs focuses on dementia diagnostics and pharmacological options, while the second workshop for nurses concentrates on dementia care issues.

#### Case-based coaching program

After attending to the workshops, GPs and nurses will select eligible patients in their own practices during the following 9 months. GPs and nurses will be coached in their performance of these patients' assessments. Three geriatric nurses will coach the participating nurses; a trained GP specialized in geriatrics will coach the participating GPs. Coaching will be performed by telephone. The coaching program is divided into two phases; in phase 1 the participants will be intensively supervised, whereas phase 2 is demand led. To gain insight into the exact content of the coaching program, coaches will keep diaries of their own performances.

#### Phase 1

##### Nurses

The first patient assessment will be discussed with a coach directly before and after completing the assessment. The second patient assessment will be performed with coaching directly afterwards. The third patient assessment will be performed without direct coaching. Diagnostic work-up and management of the first, second and third patient will be discussed with one of the coaches six weeks and three months after the nurse completed the assessment.

##### GPs

GPs will perform the assessment of the first, second and third patient on their own. Diagnostic work-up and management of the first, second and third patient will be discussed with the coach six weeks and 3 months after the GP completed the assessment.

#### Phase 2

After their participation in the workshops, and evaluation of the diagnostic work-up and management of the first three patients, GPs and nurses are expected to have been sufficiently trained to perform diagnosis and management adequately on their own. For questions during this phase, nurses and GPs will have the possibility to consult the coaches by telephone and e-mail.

#### Computerized Clinical Decision Support System (CDSS)

A CDSS is an information system designed to improve clinical decision making by using reminders [39]. Turner et al. developed and tested a CDSS especially for dementia diagnosis and management in primary care [40]. The use of this tool increased the number of dementia diagnoses reported in general practice. There was no evidence of improvement in adherence to dementia quality indicators [27]. The CCDS in the DTP has been developed for the purpose of this study. The content is based on the Dementia Guideline for Primary Care and the summarizing flow chart. It is a pro-active computer programme, which supports dementia decision making in diagnostic work-up.

### Control group

Duos in the control group will not receive the training during the trial. They will, however, be given the opportunity to attend to the DTP after the trial. This means that duos in the control group will enter the DTP with a 9 months' delay.

### Outcome measures and data collection

Primary outcome measures are the numbers of cognitive assessments and dementia diagnoses over a period of nine months, which will be measured at baseline (T0) and nine months later, at the end of the study (T1). Secondary outcomes are GPs' and nurses' attitude, confidence and knowledge regarding dementia diagnosis and management, which will be measured at baseline (T0) and nine months later, at the end of the study (T1). Other secondary outcomes are the rate of adherence to national guidelines for dementia diagnosis and management, patient satisfaction, informal carer burden and satisfaction, number of emergency calls, visits and consultations. Baseline demographic characteristics collected from GPs and nurses are: age, sex, practice experience, practice size, percentage of elderly people > 65 years and the availability of chronic disease management programs in practice. Baseline demographic characteristics collected from patients and their informal caregivers are: age, sex and co-morbidity (Table 1).

**Table 1:** Outcome measures

**Variable**	**Primary outcomes**	**Secondary outcomes**	**Background**	**Instrument/Source**	**T_0_**	**T_1_**	**T_x_**
**GPs/Nurses**							
Number of dementia diagnosis^1^		□	□	EMD^2^, self registration forms	□		□
Number of cognitive assessments^1^		□	□	EMD, self registration forms	□		□
Number of emergency consultations^1^	□		□	EMD, self registration forms	□		□
Adherence to guidelines	□		□	EMD, self registration forms/QIs	□		□
Change of knowledge	□		□	Own questionnaire			□
Change of Attitude	□		□	Own questionnaire			□
Change of skills	□		□	Own questionnaire			□
Age	□	□		Own questionnaire		□	□
Sex	□	□		Own questionnaire		□	□
Practice size	□	□		Own questionnaire		□	□
Practice experience	□	□		Own questionnaire		□	□
Percentage elderly in practice	□	□		Own questionnaire		□	□
Chronic care programs in practice	□	□		Own questionnaire		□	□

**Patients**							
Age	□	□		Own questionnaire		□	□
Sex	□	□		Own questionnaire		□	□
Co-morbidity	□	□		EMD		□	□
Satisfaction intervention	□		□	Own questionnaire	□	□	

**Informal carers**							
Age	□	□		Own questionnaire		□	□
Sex	□	□		Own questionnaire		□	□
Satisfaction intervention	□		□	Own questionnaire	□	□	
Burden informal caregiver	□		□	SSCQ^3^	□	□	

T_0 _is baseline measurement

T_1 _is follow up measurement, after 9 months

T_x _is 3 months after starting each assessment

^1 ^measurement of 'number' during a period of 9 months

^2 ^Electronic Medical Dossier

^3 ^Short Sense of Competence Questionnaire

Data on adherence to national guidelines, the number of emergency situations and the number of cognitive assessments and dementia diagnoses will be retrieved from GPs' Electronic Medical Records (EMD), from interviews with GPs and nurses and from self registration forms [41]. Self-made questionnaires will provide information on baseline characteristics, on data on GPs' and nurses' attitudes, competencies and knowledge regarding dementia diagnosis and management and on patient and informal carer satisfaction. Burden of informal carers will be assessed using the Short Sense of Competence Questionnaire (SSCQ) [42]. Data on duos' performance during the 'coaching phase' will be included in the analysis.

### Sample size calculations

We expected a change in the primary outcome measure of the incidence of dementia diagnoses and cognitive assessments from 50% to 65% of the total population of dementia. In previous studies on implementation of several other guidelines in general practice the average change was 10% [43]. However, these implementation studies focused on diseases, to which GPs already showed a high adherence to the guidelines. Because of the low adherence to dementia guidelines we saw more room for improvement and therefore we expected a change of 15% to be realistic. Cluster randomisation was taken into account when we calculated the sample size needed. In this calculation, the ratio between the number of duos in the control group and the number of duos in the intervention group was 1:2. Clusters are expected to include 5 patients on average. Intra class correlation (ICC) is expected to be 0.05 or lower. For a power of 0.80, and two-sided testing at 0.05, a total of 91 general practitioners is required.

### Statistical analysis

In addition to a confirmatory analysis using the MANOVA-method, we will also perform an exploratory analysis. The aim of the exploratory analysis is to gain more insight into possible causal relations between the rate of success of the intervention components and the outcomes. Possible mediator variables are change in knowledge, attitudes and collaboration rate. We will design a path-model, specifying hypothesized relations between predictors, mediators, moderators, and effects, and we will test its goodness of fit with the data. In this way we hope to be able to specify the most plausible interdependence pattern between the variables used in the present study.

## Discussion

In this paper we described the study design of a randomized controlled trial that evaluates the effects of a Dementia Training Program (DTP) for duos of GPs and primary care nurses. A novelty in this program is the training in fixed collaborative duos. We chose focus on collaboration, because dementia care management in collaborative interdisciplinary teams was found to be effective in improving the quality of care [31]. Another novelty in our approach is the inclusion of an individual coaching program in the DTP. This educational method appears to be highly effective modifying professional behaviour [32]. Whereas some studies used single interventions [27], we developed multifaceted intervention, since multifaceted interventions are usually more effective [33]. For the design and evaluation of the DTP, we used the MRC framework [44]. The exact content of the DTP will be described in more detail in a separate article. Methodologically strong elements in this study are the following. We avoid selection bias by computerized randomization and minimisation. Two independent blinded outcome assessors will contribute to overcoming detection bias. In addition to a confirmatory analysis, we will perform an exploratory analysis. In this way, we can clarify how certain intervention components influence the rate of success and through which pathways.

Below, we describe the design characteristics that may interfere with the reliability and validity of the future results. Firstly, the method of participant recruitment may threat the external validity of the study: GPs and nurses are free to decide whether they want to participate. This may mean that this group of participants is more interested in dementia care than their average colleagues. Therefore, they may be more motivated to learn and perform better than their non-participating colleagues would do after receiving the same training. In addition, they may already be taking better care of dementia patients and their caregivers than their non-participating colleagues. However, the possible effects of this bias would run counter to our hypotheses by negatively affecting the chances to detect differences between the experimental and the control group. These effects might affect the probability of making a type II error (incorrectly accepting H-0) but they cannot cause the making of a type I error (incorrectly rejecting H-0).

Secondly, we cannot exclude the possibility of contamination arising by chance contacts and possible knowledge exchange between GPs and nurses from different allocation conditions. We try to overcome this problem by cluster randomization: GPs from the same practices and GPs working with the same nurses are allocated to the same group.

Finally, performance bias can occur. GPs and nurses are fully aware of their assignment to either the experimental or the control group. We try to overcome this problem by reminding participants in the control group every two months of their involvement in the study.

In spite of these elements of bias, the results of this trial may provide useful information for policy makers and developers of continuing medical education. The intervention is designed according to recently reported MRC guidelines and educational standards; exploratory analysis will reveal its successful elements. In the study design, bias is avoided if possible and still its setting is one of every day practice. Dissemination of the results of this study is planned for 2009.

## Competing interests

This study was funded by "Province of Gelderland, The Netherlands" and "ZonMw: The Netherlands Organisation for Health Research and Development." MP and ID were financially supported by the funding bodies. The funding bodies did not play a role in any part of the study. The other author(s) declare that they have no competing interests.

## Authors' contributions

MP and ID were responsible for the research question. MP, ID and MOR designed the study. MP was wrote the first draft of the manuscript and was responsible for revisions. ID and MOR contributed to the drafting of the manuscript. GB gave advice on the statistical analysis. TA, ME, PL and MVD commented on the design and the manuscript. All authors read and approved the final manuscript.

## Pre-publication history

The pre-publication history for this paper can be accessed here:


